# Chemical Looping CH_4_ Reforming Through Isothermal Two-Step Redox Cycling of SrFeO_3_ Oxygen Carrier in a Tubular Solar Reactor

**DOI:** 10.3390/molecules30051076

**Published:** 2025-02-26

**Authors:** Stéphane Abanades, Xinhe Wang, Srirat Chuayboon

**Affiliations:** 1 CNRS, Processes, Materials and Solar Energy Laboratory (PROMES-CNRS), 7 Rue du Four Solaire, 66120 Font-Romeu, France; 2 State Key Laboratory of Multiphase Flow in Power Engineering, Xi’an Jiaotong University, Xi’an 710049, China; 3 Department of Mechanical Engineering, King Mongkut’s Institute of Technology Ladkrabang, Prince of Chumphon Campus, Chumphon 86160, Thailand

**Keywords:** chemical looping, methane reforming, concentrated solar energy, solar reactor, perovskite, thermochemical redox cycle, solar fuel, hydrogen, water/CO_2_ splitting

## Abstract

The chemical looping reforming of methane using an SrFeO_3_ oxygen carrier to produce synthesis gas from solar energy was experimentally investigated and validated. High-temperature solar heat was used to provide the reaction enthalpy, and therefore the methane feedstock was entirely dedicated to producing syngas. The two-step isothermal process encompassed partial perovskite reduction with methane (partial oxidation of CH_4_) and exothermic oxidation of SrFeO_3-δ_ with CO_2_ or H_2_O splitting under the same operating temperature. The oxygen carrier material was shaped in the form of a reticulated porous foam structure for enhancing heat and mass transfer, and it was cycled in a solar-heated tubular reactor under different operating parameters (temperature: 950–1050 °C, methane mole fraction: 5–30%, and type of oxidant gas: H_2_O vs. CO_2_). This study aimed to assess the fuel production capacity of the two-step process and to demonstrate the potential of using strontium ferrite perovskite during solar cycling for the first time. The maximum H_2_ and CO production rates during CH_4_-induced reduction were 70 and 25 mL/min at 1000 °C and 15% CH_4_ mole fraction. The increase in both the cycle temperature and the methane mole fraction promoted the reduction step, thereby enhancing syngas yields up to 569 mL/g during reduction at 1000 °C under 30% CH_4_ (778 mL/g including both cycle steps), and thus outperforming the performance of the benchmark ceria material. In contrast, the oxidation step was not significantly affected by the experimental conditions and the material’s redox performance was weakly dependent on the nature of the oxidizing gas. The syngas yield remained above 200 mL/g during the oxidation step either with H_2_O or CO_2_. Twelve successive redox cycles with stable patterns in the syngas production yields validated material stability. Combining concentrated solar energy and chemical looping reforming was shown to be a promising and sustainable pathway toward carbon-neutral solar fuels.

## 1. Introduction

Concentrated solar power is a renewable energy source for process heat supply to drive high-temperature thermochemical reactions, such as pyrolysis, reforming, and gasification reactions, or two-step redox cycles for fuel production. Solar energy is clean, abundant, and carbon-neutral [1,2], but it is dilute and intermittent. Thermochemical redox cycles driven with concentrated sunlight are a promising pathway to store intermittent solar energy as transportable and dispatchable chemical fuels by H_2_O or CO_2_ splitting to produce syngas. The resulting syngas (a mixture of H_2_ and CO) can be further converted to liquid hydrocarbon fuel via Fischer–Tropsch [3] or utilized for the production of methanol, ammonia, or dimethyl ether [4]. Nevertheless, the major issues of cyclic processes like these are the high reduction temperature required for a significant reduction extent (typically > 1400 °C), and the required temperature swing between the reduction and oxidation steps to enhance fuel production capacity, resulting in high sensible heat losses and leading to a decrease in solar-to-fuel energy conversion efficiency.

Alternatively, the conventional production of syngas through the reforming of CH_4_ (steam or dry reforming [5,6,7,8,9]) requires a catalyst, which may lead to catalyst deactivation issues through carbon deposition. The Ni catalysts commercially used in reforming suffer from rapid deactivation due to severe coke deposition [10]. Therefore, a steam excess (about 3–5 times the stoichiometric amount) is required in steam reforming to avoid carbon formation, thereby leading to energy losses.

Chemical looping reforming of methane (CLRM) over metal oxide oxygen carrier materials can be used to overcome such issues [11,12,13,14,15]. CLRM results in the partial oxidation of methane without the use of gaseous oxidants or catalysts, while the process can be operated as a two-step cycle at relatively low temperatures (about 800–1000 °C) thanks to the use of a gaseous reductant [16,17,18,19]. The oxygen carrier redox material is first partially reduced by methane, producing syngas (also known as the methane partial oxidation step), according to Equation (1).

MeO_x_ + δCH_4_ → MeO_x−δ_ + δCO + 2δH_2_ (1)

In a second step (Equation (2)), the reduced material is oxidized by H_2_O or CO_2_, recovering the lattice oxygen and generating pure H_2_ or CO (water or CO_2_ splitting step), while the solid oxide can be recycled back.

MeO_x−δ_ + δH_2_O/CO_2_ → MeO_x_ + δH_2_/CO (2)

The above global reactions are equivalent to conventional reforming, which consists of steam methane reforming (SMR, Equation (3)) and dry reforming of methane (DRM, Equation (4)):

CH_4_ + H_2_O → 3H_2_ + CO (3)

CH_4_ + CO_2_ → 2H_2_ + 2CO (4)

The heat required for the endothermic reaction can be supplied by solar energy using concentrating solar technologies, enabling solar energy conversion to clean and storable chemical fuels [20]. This two-step combined process (methane reforming and H_2_O/CO_2_ splitting) requires a significantly lower reduction temperature in comparison with the two-step metal oxide redox cycles [21,22], thanks to the use of a reducing gas (CH_4_) in the first step. Hence, isothermal cycling is made possible because the metal oxide reduction with CH_4_ and the H_2_O/CO_2_ splitting steps can be carried out at the same temperatures. Accordingly, the thermal constraints imposed on the reactor and oxygen carrier materials are alleviated, the need for sensible heat recovery between cycle steps is eliminated, and thermal radiation losses are reduced. Moreover, the deposited carbon can be concomitantly gasified in the H_2_O or CO_2_ splitting step before the next reduction step, and the two-step process eliminates the need for a catalyst, thereby avoiding any catalyst deactivation issues through carbon deposition that may occur in the conventional reforming process. Compared with direct combustion, the conversion of methane to syngas driven by solar energy has more favorable economics, with a reduced environmental footprint. When bio-methane is utilized as feedstock, produced syngas becomes carbon neutral. Solar energy storage as high-value syngas is achieved, for subsequent conversion into liquid hydrocarbons used as transportable and dispatchable chemical fuels. Solar chemical looping reforming allows for the valorization of methane and provides an attractive pathway for the sustainable storage of intermittent solar energy as renewable fuels.

The feasibility of using different metal oxides as oxygen carriers for CH_4_ partial oxidation has been experimentally reported, including zinc oxide [23,24,25,26,27], iron oxide [28], tungsten oxide [29], ceria (CeO_2_) [11,12,13,30], and cerium-based mixed oxides [11,31,32]. Among the potential metal oxides previously investigated, ceria has emerged as an attractive oxygen carrier candidate owing to its favorable physical and chemical properties [33,34,35], such as high oxygen storage and release capacities combined with rapid oxygen exchange rates through lattice transfer, crystallographic stability through thermochemical cycles [36], and reversible transition between Ce(IV)/Ce(III) oxidation states [37,38]. These materials, based on either doped or undoped ceria, have been shown to be attractive for the partial oxidation of CH_4_ [11,20,32,37,39,40,41,42,43]. The selection of new suitable oxygen carrier materials for solar-driven CLRM is required, and they should exhibit high redox activity and selectivity, thermal stability, and be low-cost and environmentally friendly [15]. Different single metal oxides, including Mn, Fe, Co, Ni, Cu, and Ce-based oxides, have been investigated as oxygen carrier materials for chemical looping applications [44,45,46]. Mixed oxides also attract attention, since their redox performance can be promoted via doping, compositing, or encapsulating [47,48,49]. Perovskite-based oxygen carriers represent another promising class of materials for CLRM, because they offer attractive redox properties, high oxygen exchange capacity, and a fast oxygen anion migration rate [50,51]. However, the redox activity of perovskites in solar reactors applied to CLRM has not been extensively investigated to date.

In addition, different structured materials have been considered to promote surface reactions including porous foam [34,52], macroporous structures [53], or fibers/felts [54]. The coating of ceria foam with La_0.5_Sr_0.5_Mn_0.9_Mg_0.1_O_3_ was tested for two-step CO_2_/H_2_O splitting [55], and enhanced the oxygen exchange between redox phases in the reduction step and led to increased fuel production. Oxygen carriers with porous structures like this exhibit a beneficial effect on heat and mass transfer in solar thermochemical processes. In contrast, oxygen carriers in the form of powder may induce high flow resistance, causing pressure drop, powder packing hindering the access of the reactive gases to the bulk material, and possible particle entrainment by the gas flow. Conversely, porous structures acting as the reactive material itself inherently combine the advantages of volumetric radiative absorption, low-pressure drop, high geometric surface area, rapid reaction rates, and high mass loading of the reactive material in the reactor.

Among various oxygen carrier materials, ABO_3_ perovskites exhibit remarkable redox reversibility, oxygen mobility, oxygen storage capacity, and electronic and crystal structural flexibility [56,57,58]. The A site of perovskite oxides usually contains large metal cations, such as La, Sr, and other alkali metals, which coordinate to 12 oxygen ions. The B site contains smaller cations, such as transition metals (Fe, Mn, Co, Ni, etc.), where six O atoms form a hexagonal BO_6_-type structure. Several strategies have been proposed to improve the redox performance of perovskite-type oxygen carriers [59,60]. The doping of LaFeO_3_ perovskite with Co increased the reactivity of LaFeO_3_ and inhibited methane cracking, while LaFe_0.7_Co_0.3_O_3_ showed the best performance and remained stable over 20 redox cycles [59]. High activity in solar-driven CL-SMR was observed for Fe-doped BaMnO_3_ perovskite with a syngas yield and a steam conversion approaching 90% [60].

Strontium ferrite (SrFeO_3−δ_, SFO) has been proposed as a potential material for CLRM [61]. According to thermodynamics, strontium ferrite perovskite appears to be a promising oxygen carrier for CLRM at temperatures of 800–1200 °C [61,62,63,64]. Besides favorable thermodynamic properties, it has also the advantages of being low-cost while being easily tunable. Dispersing SrFeO_3−δ_ in a CaO medium could enhance the reactivity, cyclability, and thermal stability of SrFeO_3−δ_. Zhang et al. [63] dispersed the SrFeO_3-δ_ reactive perovskite phase into a CaO support to make a mixed oxide nanocomposite, which significantly increased the redox stability and kinetics. The influence of the composition of SrFeO_3-δ_-CaO nanocomposite on CL-DRM was investigated, in order to optimize CH_4_ conversion and syngas production rate. The composition of 10 wt% SrFeO_3−δ_–CaO nanocomposite led to the maximum CH_4_ conversion (88%) and syngas production rate (1.8 mol kg_SFO_^−1^ min^−1^), whereas the composition of 80 wt% SrFeO_3−δ_–CaO nanocomposite showed the maximum syngas production (27.3 mol kg_SFO_^−1^) [64]. Wang et al. [65] studied the SrFeO_3-δ_/CaO–MnO nanocomposite as an effective oxygen carrier for chemical looping partial oxidation of methane. SrFeO_3-δ_-Ca_0.5_Mn_0.5_O nanocomposite exhibited high activity and facilitated coke gasification [66]. In the reduction step of 27 successive redox cycles, the production rate of CO changed marginally, and the CO yield was maintained at about 1.9 mmol/g while the production of H_2_ tended to be stable at 3.8 mmol/g (i.e., twice the CO yield). However, using inert supports may also induce process limitations. Indeed, dispersing the active phase in an inert supporting medium downgrades the overall fuel production capacity of the system (i.e., fuel yield per unit mass of involved material), while it also requires an additional energy supply to heat the whole structure. Moreover, chemical interactions with the support may occur. Therefore, the use of pure active phases should be preferred for enhanced process efficiency.

Modifications via partial substitution of other cations for Sr and/or Fe cations have also been considered [67,68]. Zhu et al. [69] synthesized the Sr_0.98_Fe_0.7_Co_0.3_O_3-δ_ perovskite-structured oxygen carrier while they combined both A-site defects and B-site doping of SrFeO_3-δ_. Isothermal CL-DRM experiments were conducted at 850 °C, and the material achieved CH_4_ conversion of 87% and a CO selectivity of 94% during the first step (CH_4_ partial oxidation), while a syngas yield of 8.5 mmol/g and CO yield of 4.2 mmol/g were obtained in the second step (CO_2_ splitting). In addition, the redox performance of La_0.9_Sr_0.1_FeO_3_ perovskite modified with yttria-stabilized zirconia (YSZ) was investigated during chemical looping (CH_4_ partial oxidation/CO_2_ splitting) at 850 °C, showing syngas yields of 476 μmol min^−1^ g^−1^ for H_2_ and 432 μmol min^−1^ g^−1^ for CO [70]. A CuO-modified La_0.7_Sr_0.3_FeO_3_ (LSF) was investigated to improve the redox activity of the oxygen carrier at moderate temperatures (750 °C) [68]. Finally, SrFeO_3−δ_ perovskites doped with low amounts of Co or Ni were studied as oxygen carriers for CL-DRM [71]. These doped materials had an accelerated phase transition in the first reduction step, compared with pristine SrFeO_3−δ_. Accordingly, the cation doping resulted in enhancing both the CH_4_ conversion and the amount of lattice oxygen consumption.

In this study, strontium ferrite perovskite was used as an oxygen carrier for CLRM to demonstrate the feasibility of solar fuel synthesis using an affordable and efficient redox material, without including any additional inert support. The solar process aims to produce hydrogen-rich syngas from methane reforming combined with the splitting of H_2_O and CO_2_ molecules using concentrated solar energy as the process heat source, thus reducing greenhouse gas emissions. A foam structure was synthesized by replication and cycled to evaluate the thermochemical performance in a solar-heated tubular reactor. In most previous studies, electrically-heated reactors have been used, whereas solar reactors have scarcely been developed for cycling operations under real conditions with variable incident sunlight and non-uniform heat flux conditions. In this work, the material was thus subjected to consecutive isothermal redox cycles under highly concentrated solar heating to determine its redox activity and fuel production performance during both cycle steps, including methane partial oxidation and H_2_O/CO_2_ splitting. While ceria redox materials have mostly been considered for solar-driven CLRM, the performance of a strontium ferrite perovskite material in a solar-heated reactor has never been investigated before. The solar reactor was successfully tested during continuous redox cycling of perovskite porous foam. The effect of the main operating parameters (including cycle temperature, CH_4_ mole fraction, and type of oxidant gas) on the syngas production capacity was unraveled. The redox cyclability and thermochemical stability of the material were also investigated to provide insights into the applicability of this material for solar-driven chemical looping fuel production.

## 2. Results and Discussion

The oxygen carrier material was cycled in the solar tubular reactor (mass of loaded sample: m = 1.93 g) to investigate the impact of the main operating parameters on the redox cycling performance during methane CLR. The effect of the reaction temperature, oxidant gas (H_2_O vs. CO_2_), and CH_4_ mole fraction on the gas production rates and yields was evidenced through 12 consecutive cycles. Successive cycles were carried out without changing the material to confirm that the same oxygen carrier can be cycled under various operating conditions without any significant change in the redox activity. This confirms that the reactivity is not altered due to modification of the operating conditions. Moreover, the parameters were studied in sequence, for example, cycles #3 to #5 for the effect of temperature (950–1000–1050 °C), and cycles #9 to #12 for the effect of the CH_4_ mole fraction (5–30%). Repeatability tests were also performed at the reference condition (1000 °C and 15% CH_4_) to check the results’ reproducibility. This operating condition was repeated several times to check the stability of redox performance. In particular, cycles #1, 2, 4, 6, 8, and 12 were performed under the same operating conditions to evaluate the performance of cyclic experiments. A summary of the experimental performance results is reported in Table S1 (Supplementary Materials).

### 2.1. Influence of Temperature on Syngas Production

**Figure 1:**
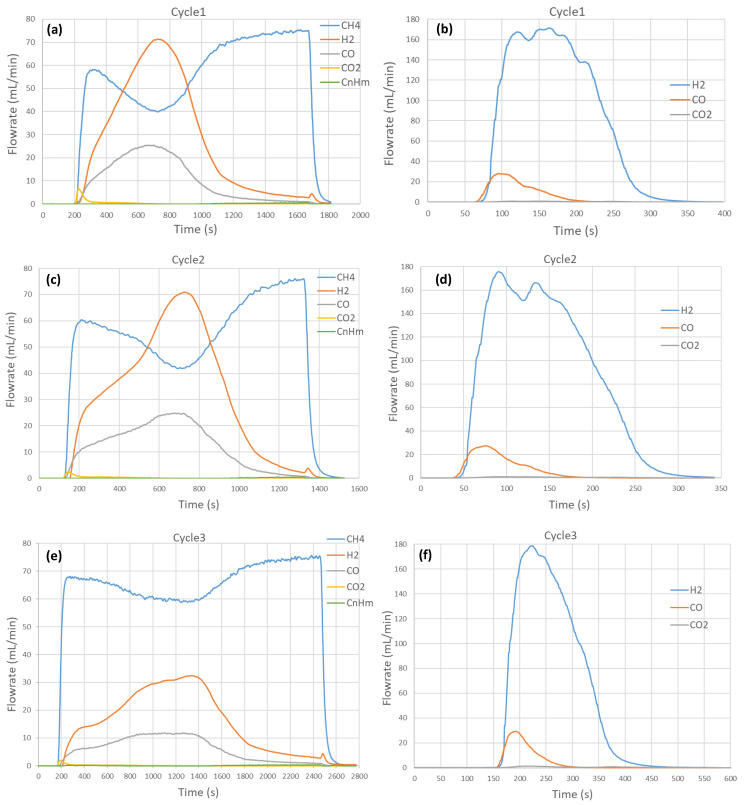

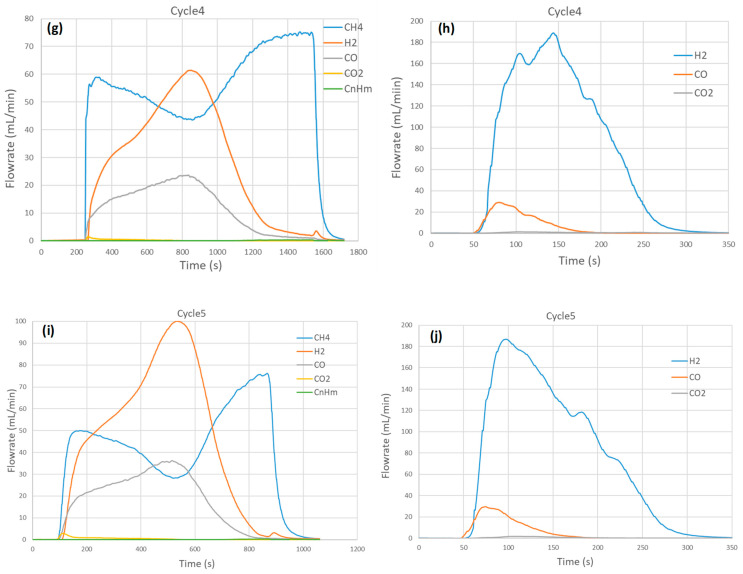
Influence of temperature on syngas production rates for CH_4_ partial oxidation and H_2_O splitting steps in (**a**,**b**) cycle #1 @ 1000 °C, (**c**,**d**) cycle #2 @ 1000 °C, (**e**,**f**) cycle #3 @ 950 °C, (**g**,**h**) cycle #4 @ 1000 °C, and (**i**,**j**) cycle #5 @ 1050 °C. Reaction conditions: $m_{SFO}$ = 1.93 g, *F*_red_ = 0.5 NL/min (15% CH_4_), *F*_ox_ = 0.736 NL/min (32.1% H_2_O).

A series of five consecutive cycles with CH_4_ partial oxidation followed by H_2_O splitting was performed to investigate the effect of temperature (950 °C, 1000 °C, 1050 °C) on syngas production capacity. Both the reduction and the oxidation steps were carried out at the same temperature through isothermal redox cycling. The obtained syngas production rates versus time profiles are reported in Figure 1. Globally, the evolution patterns are similar, which confirms the results’ reproducibility and material stability, as evidenced by cycles #1, #2, and #4 repeated at 1000 °C. During CH_4_-induced reduction (CH_4_ flowrate: 75 mL/min, with 15% mole fraction), the methane flowrate in the outlet stream increased rapidly after feeding methane into the solar reactor and then reached a maximum during the initial stage. H_2_ and CO flowrates increased promptly as soon as CH_4_ was injected, then slowed down and finally increased again to reach a peak about 8 min after CH_4_ injection at 1000 °C. The maximum H_2_ and CO flowrates were about 70 and 25 mL/min at 1000 °C (cycles #1 and #2). In the meantime, the flowrate of CH_4_ decreased and reached a minimum value when the H_2_ and CO flowrates were maximum, which denotes maximum CH_4_ conversion. Subsequently, H_2_ and CO started to decline while the CH_4_ flowrate increased again to reach the inlet value, denoting the end of the reaction. The formation of CO_2_ was detected only in the initial stage at the beginning of CH_4_ injection, due to the reaction of surface oxygen with CH_4_ according to Equation (10). The simultaneous production of both CO_2_ and H_2_O (twice the amount of CO_2_) was thus expected from complete methane oxidation, although H_2_O was not measured by gas analysis. At the end of the reaction, the H_2_ and CO production rates returned to negligible values while the CH_4_ flowrate approached the inlet value (75 mL/min), thus indicating reaction completion. The slight production of H_2_ at the end of the reaction (H_2_ flowrate not returning to zero as in the case of CO) was attributed to the CH_4_ cracking reaction forming H_2_ and solid carbon (coke) on the surface of the oxygen carrier.

It can be noted that the production of light hydrocarbons (C_n_H_m_, *n* ≥ 2) was detected in the outlet stream, but their amount remained low (3 mL in cycle #1) and such species were attributed to methane coupling reactions (nCH_4_ → C_n_H_m_ + (2n − 0.5m)H_2_). Their production occurred chiefly at the initial stage of the reduction step, when methane flowrate in the outlet stream reached the local maximum. The flowrate of light hydrocarbons then decreased smoothly and then slowly increased at the end of the reaction when H_2_ and CO declined.

During the oxidation step, H_2_O was injected (0.19 g/min, 0.24 NL/min, 32.1% in Ar) to reoxidize the partially reduced perovskite material. A sharp production of H_2_ was measured along with a moderate production of CO due to the reaction of coke with H_2_O forming both CO and additional H_2_ (Equation (11)). The production of CO_2_ (Equations (12) and (13)) remained negligible. The time to reach maximum flowrates was shorter for CO and H_2_ than for CO_2_. This delay in CO_2_ evolution can be attributed to the sequential mechanism leading to the formation of CO_2_ (carbon gasification, Equation (11), followed by a water–gas shift reaction, Equation (12)). At the early stage of the oxidation step, steam is mainly consumed by the oxidation reaction of the material, and the water–gas shift reaction remains negligible. At the late stage of the oxidation step, steam is in excess and thus the shift reaction is favored. The maximum H_2_ flowrate reached 170 mL/min in cycles #1 and #2 and increased to over 180 mL/min in other cycles, whereas the CO flowrate remained below 30 mL/min in all the cycles. Moreover, the CO flowrate decreased to zero faster than the H_2_ flowrate. This means that the carbon gasification (Equation (11)) was faster than the perovskite oxidation with H_2_O producing H_2_ (Equation (6)).

A significant impact of temperature on syngas production rates and reaction duration was noticed and was more pronounced for the reduction than for the oxidation step. The maximum H_2_ flowrate in the first step increased from 31 mL/min at 950 °C (cycle #3) to 100 mL/min at 1050 °C (cycle #5), while the CO flowrate also increased moderately from 11 to 36 mL/min. The maximum CH_4_ conversion (ratio of CH_4_ converted to CH_4_ in the feed gas) also increased with the temperature from 21.5% at 950 °C (cycle #3) to 62.4% at 1050 °C (cycle #5). The duration of the reduction step was also drastically shortened when the temperature was increased (13 min at 1050 °C vs. >35 min at 950 °C). In contrast, the oxidation step duration was not drastically influenced by the temperature (5 min at 950 °C vs. 4 min at 1050 °C).

**Figure 2:**
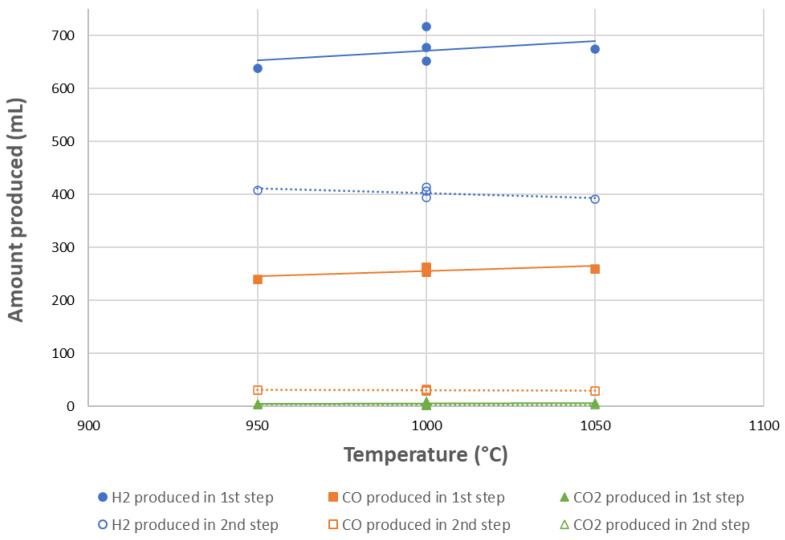
Influence of temperature on syngas production yields for CH_4_ partial oxidation (1st step, solid lines) and H_2_O splitting (2nd step, dotted lines) at 950 °C (cycle #3), 1000 °C (cycles #1, 2, 4), and 1050 °C (cycle #5).

Figure 2 illustrates the influence of temperature on the gas production yields for cycles #1 to #5. Globally, the H_2_ and CO yields in the CH_4_ reduction step increased slightly with temperatures between 950 and 1050 °C, whereas the H_2_ and CO yields in the second oxidation step remained stable. The H_2_ yield increased from 639 mL at 950 °C to 675 mL at 1050 °C, while the CO yield rose from 240 mL to 260 mL in the first step. A higher syngas yield was observed during the first cycle at 1000 °C, presumably due to the fresh material not being cycled, resulting in the absence of coke deposit on the surface, which is associated with a higher reactivity. The total H_2_ and CO yields including both steps were 1046 and 270 mL at 950 °C, increasing to 1065 and 289 mL at 1050 °C, respectively. The total CO_2_ yield remained low (e.g., up to about 9 mL at 950 °C and 1050 °C).

### 2.2. Influence of the Oxidizing Agent in Second Step on Syngas Production

**Figure 3:**
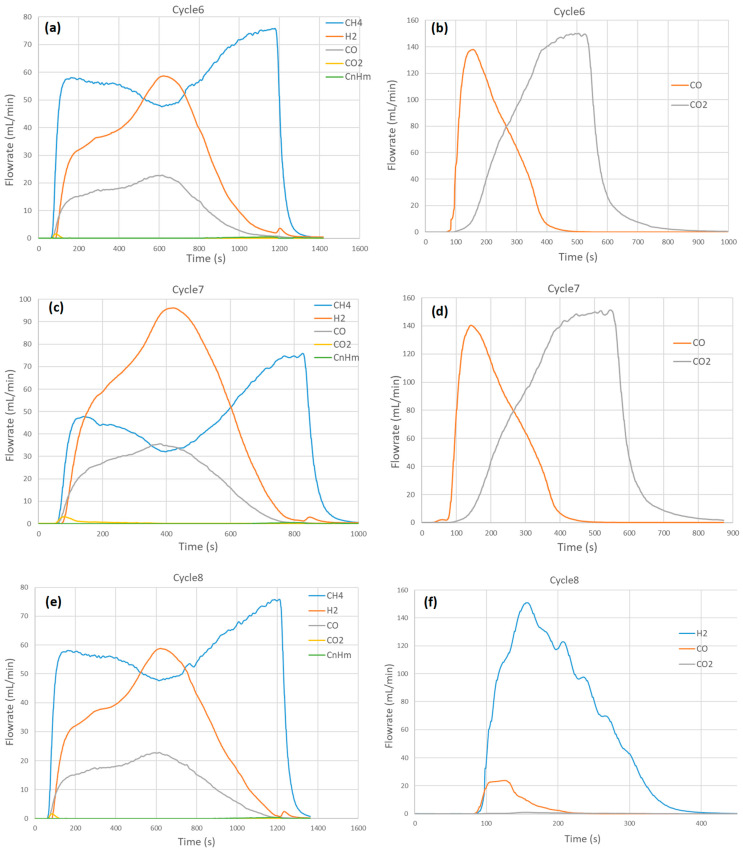
Influence of the oxidizing agent on syngas production rates for CH_4_ partial oxidation and CO_2_/H_2_O splitting steps in (**a**,**b**) cycle #6 (@ 1000 °C with 30% CO_2_), (**c**,**d**) cycle #7 (@ 1050 °C with 30% CO_2_), and (**e**,**f**) cycle #8 (@ 1000 °C with 32% H_2_O). Reaction conditions: $m_{SFO}$ = 1.93 g, *F*_red_ = 0.5 NL/min (15 vol.% CH_4_).

The oxidation step was carried out under CO_2_ in cycles #6 and #7 to investigate the influence of the oxidizing agent during the second step. The CO_2_ mole fraction was fixed at 30% (150 mL/min CO_2_ in Ar) to allow comparison with the other cycles carried out under H_2_O at a similar steam mole fraction (for instance, cycle #8). These cycles were performed at 1000 °C and 1050 °C with 15% CH_4_ (75 mL/min) in the reduction step. Figure 3 provides the syngas production rate profiles in both steps from cycles #6 to #8. During the reduction step, the maximum H_2_ flowrate was reached in cycle #7 (over 96 mL/min, which is similar to cycle #5 at the same temperature, confirming data repeatability). Similarly, the CO flowrate reached a maximum in cycle #7 at 36 mL/min (similar to cycle #5). The amount of syngas produced in the reduction step increased slightly due to the different temperatures between cycles #6 and #7. As a result, the amount of CO produced in the oxidation step was also enhanced from 414 mL in cycle #6 to 433 mL in cycle #7. At the initial stage, CO_2_ conversion was almost close to 100% as the CO_2_ flowrate was near zero while the CO flowrate was increasing steeply. As the oxidation was proceeding after reaching the peak in CO production, the CO_2_ conversion dropped rapidly as the CO_2_ flowrate increased.

The oxidation step’s total duration was about 5 min regardless of the oxidation temperature corresponding to the period of CO detection. CO production started as soon as CO_2_ was injected, and the CO flowrate promptly reached a peak (at 140 mL/min). In the meantime, the CO_2_ flowrate increased steadily to reach the inlet value (150 mL/min) when the reaction was complete, as confirmed by the CO flow rate returning to zero.

Due to coke formation in the reduction step, carbon gasification by CO_2_ (C + CO_2_ → 2CO) likely occurred simultaneously in the oxidation step. The oxidation behaviors between CO_2_ and H_2_O oxidants were similar because the concentration of CO_2_ was very close to that of H_2_O (30 vol.% vs. 32 vol.%). Similar H_2_ and CO production rate patterns were achieved with close kinetic rates. This suggests that the chemical reaction of oxidizing gases (CO_2_ or H_2_O) at the surface was not a rate-controlling step in the oxidation step.

**Figure 4:**
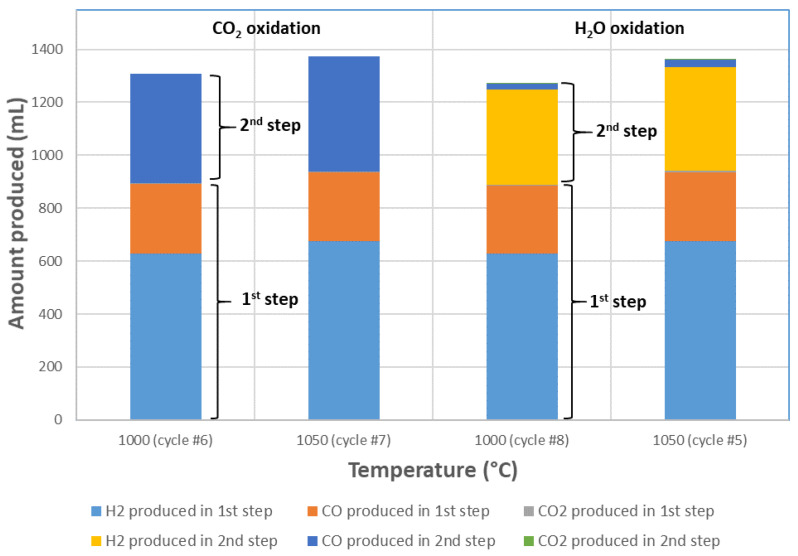
Influence of the oxidizing agent (CO_2_ or H_2_O) on syngas production yields at 1000 °C and 1050 °C (reduction step under 15% CH_4_).

Figure 4 compares the syngas yields in both steps for cycles performed at 1000 °C and 1050 °C with either H_2_O or CO_2_ during oxidation (following a same reduction step under CH_4_). In comparison with the cycles performed with H_2_O, the oxidation with CO_2_ only produced CO (mixed with unconverted CO_2_), whereas oxidation with H_2_O produced H_2_ and CO (due to reaction with carbon, Equation (11)). However, the total amount of syngas produced including both cycle steps remained stable at a given temperature (e.g., 1363 mL in cycle #5 with H_2_O vs. 1373 mL in cycle #7 with CO_2_ at 1050 °C).

### 2.3. Influence of CH_4_ Mole Fraction in First Step on Syngas Production

**Figure 5:**
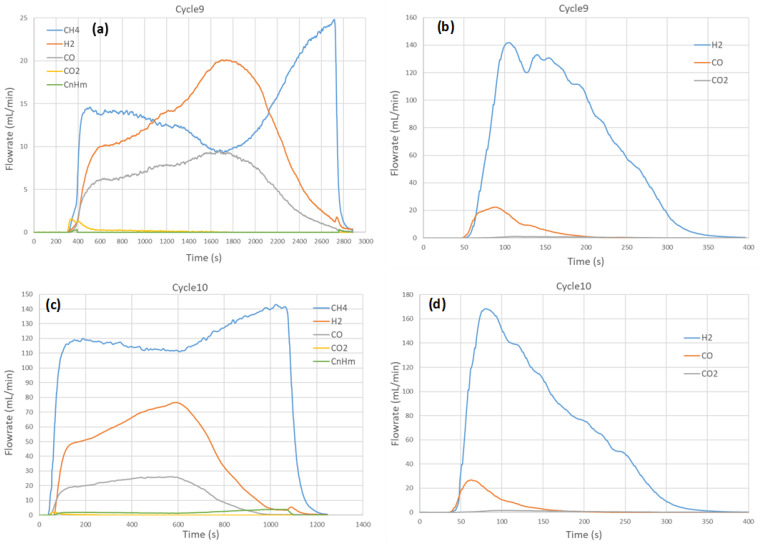

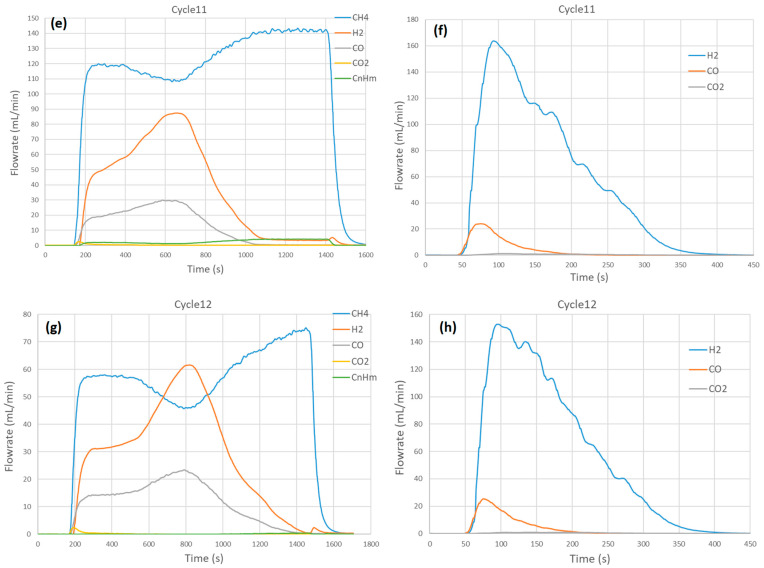
Influence of CH_4_ mole fraction on syngas production rates for CH_4_ partial oxidation and H_2_O splitting steps at 1000 °C in (**a**,**b**) cycle #9 (5% CH_4_), (**c**,**d**) cycle #10 (30% CH_4_), (**e**,**f**) cycle #11 (30% CH_4_), and (**g**,**h**) cycle #12 (15% CH_4_). Reaction conditions: $m_{SFO}$ = 1.93 g, *F*_red_ = 0.5 NL/min, *F*_ox_ = 0.736 NL/min (32.1% H_2_O).

Different cycles were carried out to investigate the impact of CH_4_ mole fraction on syngas production capacity at a constant cycling temperature of 1000 °C. The experimental production rate profiles are represented in Figure 5 for cycles #9 to #12. The CH_4_ mole fraction was varied while keeping constant the global inlet gas flowrate (at 0.5 NL/min). The corresponding inlet CH_4_ flowrate was 25 mL/min (5% in cycle #9), 75 mL/min (15% in cycle #12), and 150 mL/min (30% in cycles #10-11). The maximum CH_4_ conversion was about 63% in cycle #9, 27% in cycles #10-11, and 39% in cycle #12. The CH_4_ conversion thus increased when the methane flowrate or mole fraction decreased. The syngas production rate increased consistently with the increase of the CH_4_ flowrate. Indeed, the maximum H_2_ flowrate produced was 20 mL/min at 5% CH_4_, and it increased to 61 mL/min at 15% CH_4_ and 88 mL/min at 30% CH_4_. The CO flowrate increased from 9 mL/min to 23 and 30 mL/min, respectively. During the oxidation step, the syngas flowrate also increased, from 22 and 142 mL/min for CO and H_2_ at 5% CH_4_ to 23 and 163 mL/min at 30% CH_4_. The total duration of the reduction step was also strongly affected by the CH_4_ mole fraction. It dropped from 41 min to 15 min when the CH_4_ mole fraction increased from 5% to 30%. Similarly, the oxidation duration slightly decreased from 5 min to 4 min, respectively.

**Figure 6:**
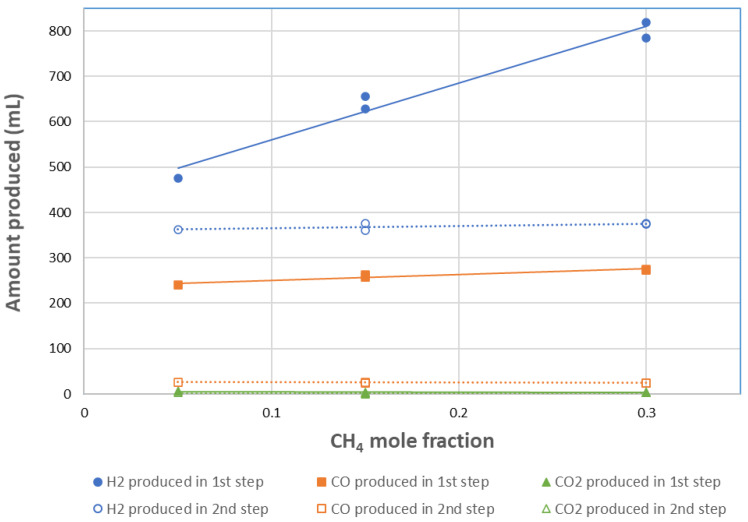
Influence of CH_4_ mole fraction on syngas production yields for CH_4_ partial oxidation (1st step, solid lines) and H_2_O splitting (2nd step, dotted lines) at 1000 °C in cycle #8 (15% CH_4_), cycle #9 (5% CH_4_), cycle #10 (30% CH_4_), cycle #11 (30% CH_4_), and cycle #12 (15% CH_4_).

Figure 6 shows the influence of CH_4_ mole fraction on the gas production yields for cycles #8 to #12. Globally, the H_2_ and CO yields in the CH_4_ reduction step increased with CH_4_ mole fraction between 5% and 30%. It can be noticed that the CO amount produced increased slightly from 240 mL at 5% CH_4_ to 275 mL at 30% CH_4_ (representing a 15% increase), whereas the H_2_ volume increased more steeply from 475 to 819 mL, respectively (corresponding to a 72% increase). Therefore, the yield of CO varied slightly compared to H_2_ as the methane flowrate/mole fraction increased, although the maximum production rate of CO increased. In fact, the rise in the maximum syngas production rate was caused by the rapid partial oxidation rate at high partial pressures of methane. Conversely, the total CO yield depends mainly on the amount of available lattice oxygen involved in the reduction step, which does not rely on CH_4_ feeding conditions provided that the reduction step has reached completion. Thus, this explains why the yield of CO remains nearly the same. The yield of syngas increases as the methane flowrate increases, chiefly because the H_2_ yield in the first step is enhanced, which is mainly due to methane cracking and coupling rather than partial oxidation. The amount of coke formed in the reduction step is, however, not directly related to the methane flowrate, because it depends on the relative rate of methane cracking to carbon gasification (with H_2_O or CO_2_). No clear trend can thus be drawn. When the reaction conditions change, more coke is formed only if methane cracking becomes faster than coke gasification. Thus, the relative rate of coke formation is dependent on the various oxidation reactions involving carbon and occurring simultaneously.

Regarding H_2_O splitting, the H_2_ and CO yields in the second oxidation step remained stable. Moreover, the results’ reproducibility was confirmed as the obtained syngas yields were similar for the two cycles performed repeatedly at 15% and 30% of CH_4_.

In summary, it appeared that the effect of the methane flowrate on the syngas (H_2_ and CO) production rate was significant, because the reduction rate is affected by the CH_4_ partial pressure. In contrast, based on the fact that the higher H_2_ yield in the first step was just due to higher methane cracking, the total yield produced from redox reactions with the perovskite oxygen carrier (Equations (5) and (6)) remained unchanged. This is because both the formation and replenishment of lattice oxygen reached a threshold value that depends on the cycling temperature and partial pressure of water, which were maintained unchanged in these redox cycles.

### 2.4. Thermochemical Cycling Stability and Materials Characterization

**Figure 7:**
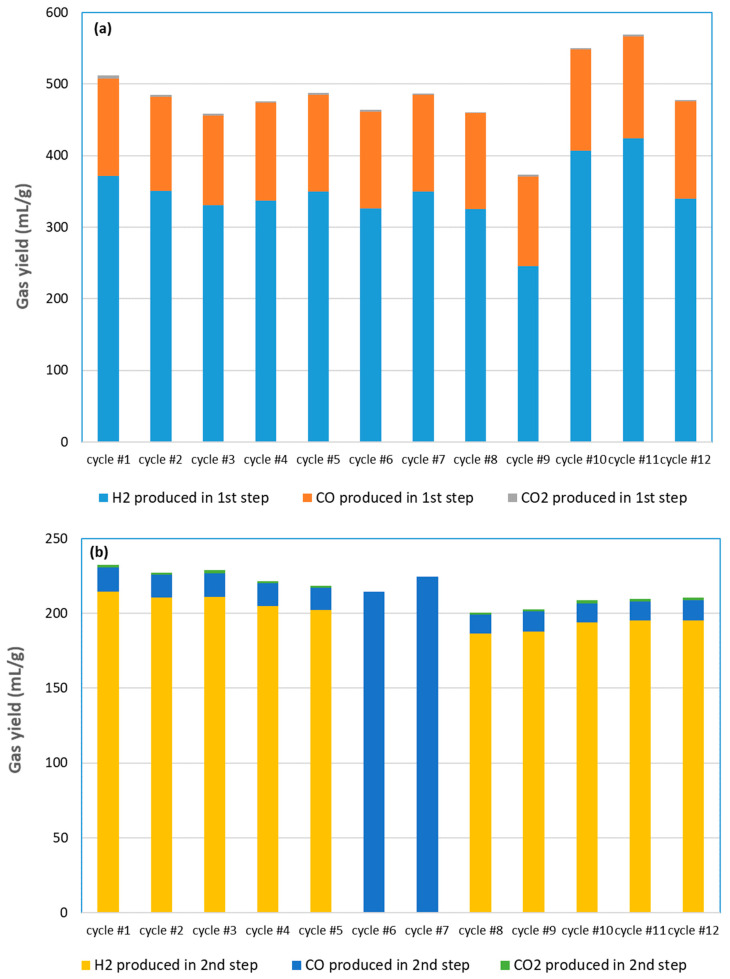
Thermochemical performance of SFO foam during 12 consecutive cycles: (**a**) syngas yields in CH_4_ partial oxidation (1st step) and (**b**) syngas yields in H_2_O/CO_2_ splitting steps (2nd step).

Figure 7 illustrates the redox performance of SrFeO_3_ foam in 12 successive cycles under the corresponding experimental conditions. It provides an overview of the syngas production yields in both steps (expressed in mL/g of oxide), which aims to show the evolution of redox performance through successive cycles. In the methane partial oxidation step (Figure 7a), the total syngas yield fluctuates in the range 464–512 mL/g when using 15% CH_4_ (cycle #1 to cycle #8, and cycle #12), except in cycle #3, which was carried out at 950 °C, leading to a slightly lower yield (458 mL/g). The lowest syngas yield of 374 mL/g was obtained at 5% CH_4_ (cycle #9), whereas the syngas yield was in the range of 550–569 mL/g under 30% CH_4_ (cycle #10 and cycle #11). Moreover, the CO yield in the reduction step was stable between cycle #1 and cycle #12 at ~136 mL/g, indicating remarkable stability in 12 cycles, without any loss in the oxygen exchange capacity. The yield of H_2_ was more influenced by the experimental conditions (both temperature and CH_4_ mole fraction) than the CO yield. It was slightly higher in the first cycle (371 mL/g) than in subsequent cycles, because the fresh material was not subjected to any cycle and coke deposition. In comparison with the CO yield, the higher H_2_ yield is attributed to methane cracking or coupling. The resulting H_2_ to CO ratio is thus higher than two (2.7 in cycle #1, decreasing to 2.4–2.6 in the next cycles). The amount of CO_2_ produced is also higher in the first cycle (~4.7 mL/g) and then decreases to lower values (~1.9 mL/g in cycle #12).

During the oxidation step for H_2_O splitting (Figure 7b), the H_2_ production yield slightly decreases from 215 mL/g in cycle #1 to 187 mL/g in cycle #8, and then slightly increases and remains almost constant as the cycle number increases (195 mL/g in cycle #12). In addition, the average CO production yield does not change significantly (in the range 13–16 mL/g). The decrease in H_2_ yield may likely be attributable to sintering, in addition to coke deposition. In the oxidation step, the total yield of syngas (H_2_O splitting step) remains stable at about 207 mL/g after 12 cycles. Moreover, the yield of CO achieved during the CO_2_ splitting step (cycles #6 and #7) is also similar and reached up to 224 mL/g in cycle #7 at 1050 °C. This demonstrates that the material can be used for both H_2_O and CO_2_ splitting without altering its redox performance, thus warranting the material’s flexibility upon cycling under different conditions.

The repeatability of syngas production performance was also validated through cycles performed under identical operating conditions: for instance, cycle #8 compared to cycle #12 (at 1000 °C and 15% CH_4_ mole fraction) and cycle #10 compared to cycle #11 (at 1000 °C and 30% CH_4_ mole fraction).

In comparison, cycling experiments have previously been carried out with ceria porous foams at 1000 °C [34]. As a result, during the reduction step, the H_2_ (CeO_2_ + CH_4_), CO, and CO_2_ yields were stable, ranging between 3.39 and 3.68 mmol/$g_{{CeO}_{2}}$ for H_2_ (75.9–82.4 mL/g), 1.69 and 1.84 mmol/$g_{{CeO}_{2}}$ for CO (37.9–41.2 mL/g), and 0.05 and 0.07 mmol/$g_{{CeO}_{2}}$ for CO_2_ (1.1–1.6 mL/g). During oxidation (CeO_2−δ_+ H_2_O), the H_2_ yield was fairly stable (1.94–2.05 mmol/$g_{{CeO}_{2}}$, 43.5–45.9 mL/g). Thus, the performance of SrFeO_3_ outperformed those of ceria (by a factor of about x4) under similar operating conditions. In another work [52], cycling experiments with ceria foam were carried out with both steps performed at 1000 °C (CH_4_ flowrate of 0.2 NL/min during reduction and H_2_O flowrate of 0.2 g/min during oxidation). As a result, the total yields of H_2_ and CO during cycling were stable (5.55–5.80 and 1.74–1.82 mmol/$g_{{CeO}_{2}}$, respectively), thus resulting in an almost constant H_2_/CO ratio (3.13–3.2), while the total syngas yield (7.55–7.89 mmol/$g_{{CeO}_{2}}$; i.e., 169.1–177.5 mL/g) was significantly lower compared to this study, thus confirming a higher oxygen release and syngas production with SrFeO_3_ than with CeO_2_. Thus, this perovskite material enhances fuel production capacity and can be cycled without any significant performance loss.

**Figure 8:**
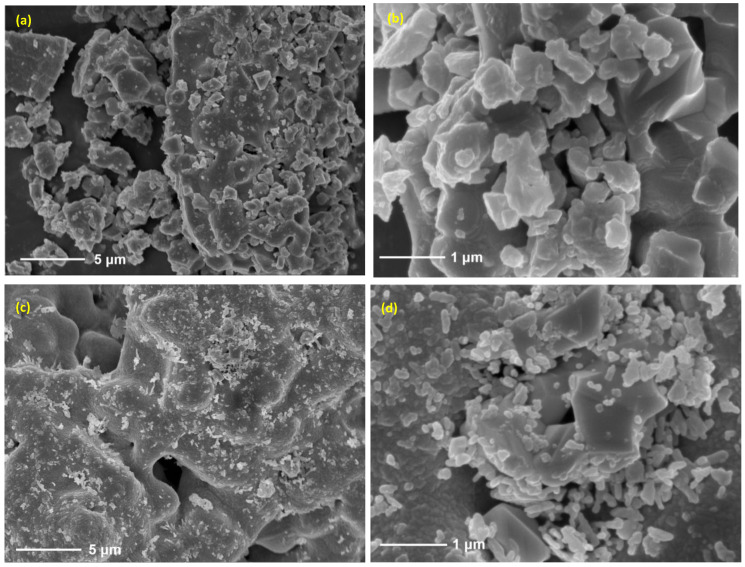
SEM analysis of SFO material before (**a**,**b**) and after 12 chemical looping reforming cycles (**c**,**d**).

The crystalline phase of the material and the foam porous structure also remained unchanged after redox cycles. According to XRD analysis, the initial material’s stoichiometry was SrFeO_2.75_ while it was SrFeO_2.5_ after the performed cycles (Figure S1). XRD then confirmed that the non-stoichiometric perovskite phase is preserved during cycles. These observations suggest that the stability and cyclability of the perovskite foam during CLRM is maintained after the performed redox cycles. As shown in Figure 8, the global porous morphology is not altered by the solar-driven thermochemical reactions, and the average particle size of the material is not significantly changed after 12 redox cycles, which suggests that sintering is not significant.

The shaping of the reactive material as a reticulated porous structure with high geometrical surface area was thus beneficial for enhancing syngas production. Surface reactions indeed benefit from active materials with easily accessible pores and high surface areas to promote oxygen transport and exchange properties. Key challenges remain in scaling up this technology for industrial applications, including the design of a suitable large-scale solar reactor integrating the porous oxide foam, and the synthesis of large amounts of perovskite oxygen carrier in the form of reticulated porous structures.

## 3. Experimental Set-Up, Materials, and Methods

### 3.1. Principle of the Chemical Looping Process, Involved Reactions, and Quantification of Product Yields

The chemical looping reforming process encompasses two consecutive steps. In the first endothermic step, the reaction of partial oxidation of methane (or partial perovskite reduction) producing syngas can be written as:

(5) $${SrFeO}_{3}+\delta CH_{4}\to SrF{eO}_{3-\delta }+\delta CO+2{\delta H}_{2}$$

In the second exothermic step, the oxygen-deficient perovskite can then react with either H_2_O or CO_2_ to produce additional H_2_ or CO:

(6) $$SrFeO_{3-\delta }+{\delta H}_{2}O\to {SrFeO}_{3}+\delta H_{2}$$

(7) $$SrF{eO}_{3-\delta }+\delta CO_{2}\to {SrFeO}_{3}+\delta CO$$

The global net reaction thus corresponds to either steam or dry methane reforming. Possible secondary reactions may occur during the reduction step with methane:

(8) $$CH_{4}\to C+2H_{2}$$

(9) $${SrFeO}_{3}+\delta C\to SrF{eO}_{3-\delta }+\delta CO$$

Other possible reactions are the oxide reduction with H_2_ and CO (formed from Equation (5)) yielding H_2_O and CO_2_ (reverse of Equations (6) and (7)), with the net reaction as follows:

(10) $${4SrFeO}_{3}+\delta CH_{4}\to 4SrF{eO}_{3-\delta }+\delta {CO}_{2}+2{\delta H}_{2}O$$

During perovskite oxidation with H_2_O (2nd step), side reactions related to the carbon deposition in the first step (Equation (8)) are expected:

(11) $${C+H_{2}O\to CO+H}_{2}$$

(12) $${CO+H_{2}O\to CO_{2}+H}_{2}$$

The sum of the above reactions corresponds to:

(13) $${C+{2H}_{2}O\to CO_{2}+2H}_{2}$$

When the perovskite is oxidized with CO_2_, the Boudouard reaction can occur as a potential side reaction associated with the carbon deposition in the first step:

(14) $$C+CO_{2}\to 2CO$$

The amount of hydrogen produced from reaction (5) is obtained from the total amount of hydrogen measured in the first step minus the amount of H_2_ produced by CH_4_ cracking (CH_4_ → C + 2 H_2_). It is assumed that the H_2_ coming from the formation of C_n_H_m_ (nCH_4_ → C_n_H_m_ + (4n − m)/2 H_2_) is negligible.

(15) $$n_{H_{2}}=n_{H_{2},total}-n_{H_{2},cracking}$$

(16) $$n_{H_{2},cracking}=n_{H_{2},total}-2n_{CO}$$

The amount of carbon (coke) produced by CH_4_ cracking can then be deduced:

(17) $${n_{C}=n}_{H_{2},cracking}/2=n_{H_{2},total}/2-n_{CO}$$

The amount of C can also be calculated from the amount of CO and CO_2_ produced in the second step (Equations (11) and (12)):

(18) $$n_{C}=n_{CO}+n_{{CO}_{2}}$$

During partial reduction with methane (Equation (5)), oxygen is released from the oxide lattice and is then recovered at the outlet in the form of CO, CO_2_, and H_2_O. Therefore, the amount of oxygen released from the oxide (oxygen consumed) can be calculated by:

(19) $$n_{O,red}=n_{CO}+{2n}_{{CO}_{2}}+n_{H_{2}O}$$

where n_i_ are the mole amounts of species i.

During the oxidation step with H_2_O (Equation (6)), oxygen is replenished in the oxide and the amount of oxygen recovered can be calculated from the total amount of H_2_ produced minus the amounts of H_2_ produced by the reactions of carbon with H_2_O (equal to the amount of CO and twice the amount of CO_2_) (Equations (11)–(13)):

(20) $$n_{O,ox}=n_{H_{2}}-n_{CO}-{2n}_{{CO}_{2}}$$

In the case of an oxidation step with CO_2_ (Equation (7)), the amount of oxygen recovered can be calculated using a mass balance on oxygen:

(21) $$n_{O,ox}={2n_{{CO}_{2},in}-n}_{CO,out}-2n_{CO_{2},out}$$

When carbon deposition occurs, the amount of additional CO produced from Equation (14) (equal to the total outlet CO minus the CO produced from Equation (7)) can be quantified separately $\left(=n_{CO,out}-n_{O,ox}=2n_{CO,out}+2n_{{CO}_{2},out}-2n_{{CO}_{2},in}\right)$. The amount of reacted CH_4_ can be calculated as follows:

n_CH4,reacted_ = n_CO,red_ + n_CO2,red_ + n_CO,oxi_ + n_CO2,oxi_ (22)

Syngas and coke selectivity are calculated as follows,

(23) $$S_{Syn}\left(\%\right)=\frac{\int_{0}^{t_{red}}F_{CO,red}dt+\int_{0}^{t_{red}}F_{H_{2},red}dt}{3(t_{red}F_{{CH}_{4},i}-\int_{0}^{t_{red}}F_{{CH}_{4},out}dt)}$$

(24) $$S_{coke}\left(\%\right)=\frac{n_{coke}}{t_{red}F_{{CH}_{4},i}-\int_{0}^{t_{red}}F_{{CH}_{4},out}dt}$$

where $F_{{CH}_{4},out}$, $F_{H_{2},red}$, and $F_{CO,red}$ are the molar flowrates of output CH_4_, H_2_, and CO, respectively. $F_{{CH}_{4},i}$ is the molar flowrate of input methane. t_red_ is the duration of the reduction step. n_coke_ is the total mole amount of coke, which is calculated using the amount of CO and CO_2_ in the H_2_O splitting step. S_syn_ and S_coke_ are syngas selectivity and coke selectivity, respectively.

### 3.2. Materials Synthesis and Characterization

The detailed procedure of preparing SFO powder has been described previously [37]. The material was shaped in the form of a porous foam structure made of the redox material itself, thus avoiding the use of any additional inert support. The SFO powder was ground to less than 20 μm and then mixed with the organic adhesive polyvinyl butyral (Butvar B-98). The mass ratio of SFO to Butvar B-98 was 5:1. The mixture was then placed in ethanol. The mass ratio of SFO powder to ethanol was 2:1. Afterwards, the slurry was agitated (300 rpm) at 60 °C until a suspension was obtained, followed by cooling down to room temperature. A polyurethane template of 10 ppi (pore per inch) was used and shaped into a cylinder to match the reactor tube. The template was dipped into the suspension several times to obtain sufficient SFO coating. The resulting coated template was first dried at room temperature for 20 min and then calcined for 6 h at 1000 °C. The oxygen carrier was thus shaped as a reticulated porous ceramic (RPC) structure with a well-connected and open porosity, offering high surface-to-volume ratio for solid–gas reactions and favorable heat and mass transfer.

The crystalline phase of the samples was examined by X-ray diffraction (XRD) using a Shimadzu XRD-6100 (Shimadzu, Kyoto, Japan) diffractometer, equipped with a Cu Kα radiation source (λ = 0.154056 nm) at the operating power of 2.2 kW. The samples were scanned over a 2θ range of 8–90° with a scan rate of 6°/min at ambient conditions. The morphology of the samples was examined by scanning electron microscopy (SEM) on a TESCAN MAIA3 LMH (TESCAN, Brno, Czech Republic) operated with an accelerating voltage of 5–15 kV. Samples for SEM were deposited onto an adhesive conductive carbon belt attached to the sample holder.

### 3.3. Solar Tubular Reactor

**Figure 9:**
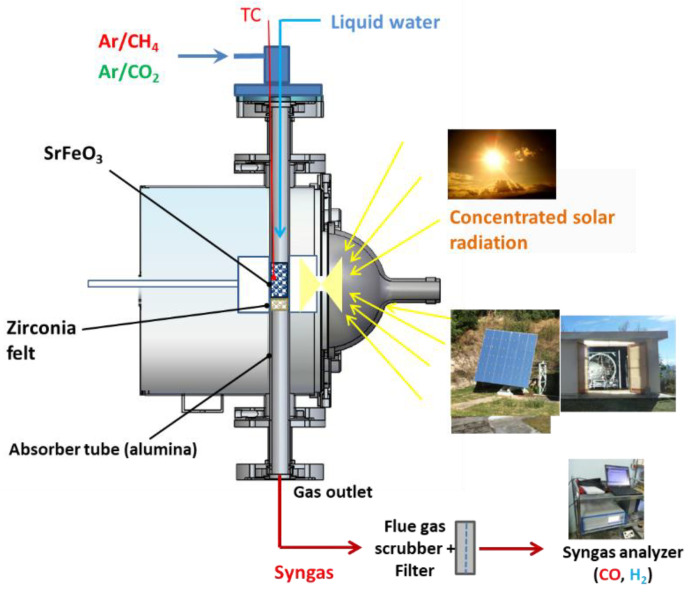
Scheme of the windowed solar tubular reactor for two-step chemical looping reforming with solid oxygen carrier materials as a reticulated porous foam inside the vertical tube.

**Figure 10:**
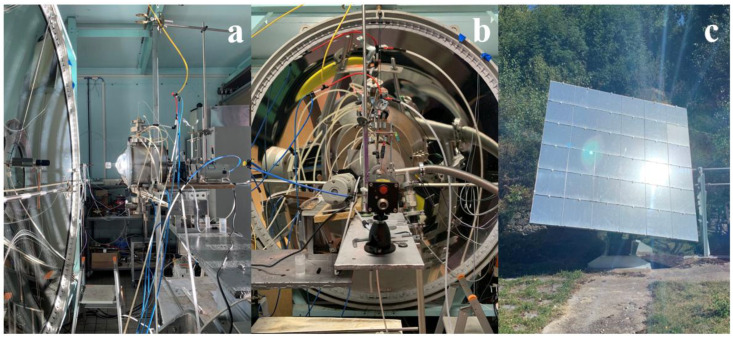
Pictures of (**a**) solar reactor during on-sun operation, (**b**) parabolic dish solar concentrator and reactor installed, and (**c**) sun-tracking heliostat.

Cycling experiments for chemical looping methane reforming were carried out in an indirectly irradiated packed-bed tubular solar reactor at the focus of a solar concentrator (Figure 9). The solar thermochemical reactor was developed to investigate various high-temperature gas–solid reactions under controlled atmospheres [37,72,73]. This reactor was settled at the focal point of a medium-size horizontal-axis solar furnace, comprising a sun-tracking heliostat that horizontally reflects the incident solar radiation flux towards a concentrator (2 m-diameter parabolic dish), thereby supplying the required high-temperature solar heat (Figure 10). Real concentrated solar energy thus provided the high-temperature process heat used as the energy source for driving the isothermal chemical looping redox process. The solar reactor mainly consisted of a cavity-type solar receiver (made of graphite), with a front disc aperture (20 mm-diameter) settled at the focus of the solar concentrator to let concentrated solar energy enter and be absorbed. The cavity was surrounded by thermal insulation and the reactor was tightly closed by a transparent Pyrex glass window fixed at its front face. The nominal concentrated solar power absorbed within the cavity via the front aperture reached ~1.5 kW (with a Direct Normal Irradiation-DNI of 1 kW.m^−2^). The oxygen carrier porous foam material was placed at the center of an alumina tube (20 mm internal diameter, 25 mm external diameter) crossing the solar absorber cavity vertically and was supported by an inert support layer (3 mm-thick disc) made of porous zirconia felt (Zircar Inc., Florida, NY, USA) that is stable and chemically inert under the operating conditions to avoid unwanted side reactions. The solar heat was transferred to the redox material via the alumina tube (indirect heating). This cavity-type configuration provides homogeneous temperature distribution along the tubular reacting zone (40 mm height), where the sample is placed.

The reactor was first flushed with inert gas (0.2 NL/min of pure argon, 99.999% Ar purity, [O_2_] < 2 ppm) to remove residual air in the tube and was then heated to the targeted cycle temperature. A shutter positioned on the path of the reflected sunlight was used to control the solar energy input, thereby adjusting the operating temperature of the cycle by controlling the shutter opening in real time. Once the isothermal conditions were reached, the cycle was started. During the reduction step under solar heating, CH_4_ (mixed with Ar at a total gas flowrate of 0.5 NL/min to keep the gas residence time constant) was injected through the tube to react with the oxide (partial oxidation step) for the production of syngas. When the amount of CO was close to zero, methane injection was stopped, and then Ar purging was kept until the baseline signals of gases stabilized. The oxidation step (with either H_2_O or CO_2_) of the oxygen-deficient material was then performed at the same temperature under solar heating to produce H_2_ or CO. When the signal of H_2_ or CO was close to zero, the feeding of water or CO_2_ was stopped, followed by purging with Ar until the signals of gases were stable. Then, the next redox cycle could be started.

The reaction temperature was directly measured by a K-type thermocouple inside the reacting sample. In addition, a solar-blind pyrometer (Heitronics KT15, 4.9–5.5 µm, Wiesbaden, Germany) pointing to the tube external wall was used to measure the tube temperature at the external wall side directly exposed to concentrated radiation through a CaF_2_ window. The inlet gas flow rate (Ar carrier gas with 99.999% purity, CH_4_ with 99.95% purity, CO_2_ with 99.995% purity) and liquid H_2_O flowrate (deionized water) were regulated using electronic mass flow controllers (Brooks Instruments, Hatfield, PA, USA, range 0–2 NL/min, precision ±0.2% of full scale for CH_4_ and CO_2_, range 0–30 g/h ± 1% of full scale for H_2_O). The flowrate of H_2_O was set at 0.19 g/min (32.1% in 0.5 NL/min Ar) and the volume fraction of CO_2_ was 30% at a total flowrate of 0.5 NL/min. Water was injected into the tube from the top above the material via a stainless-steel capillary and was vaporized when exiting the hot capillary tube. Steam was then entrained by the inert gas and flowed through the reactive material to oxidize it and produce H_2_. All the gases flowed from the top to the bottom of the tube and the outlet gas flowed through a gas drying unit (bubbler and desiccant column to eliminate excess steam) and filter (pore diameter of 0.1 μm) prior to gas products analysis. Then, the outlet products (syngas) were transported by the carrier gas to an online gas analyzer (GEIT 3100, Pollutek, Pellenberg, Belgium, uncertainty ≤ 2% FS) for continuous measurement of H_2_, CO, CO_2_, CH_4_, and C_n_H_m_ concentrations. A gas chromatograph was also used for complementary analysis (micro-GC, Varian CP4900, Palo Alto, CA, USA), for data comparison and validation.

The gas production yields were then quantified by time integration of the gas production rates. All the physical data measurements were acquired by an automated data acquisition system every 1 s. Based on the uncertainty of gas analysis systems, the relative uncertainty for the calculation of the syngas yields and other reactor performance metrics was found to be ±2.5%. The detailed operating conditions of each cycle are listed in Table 1.

## 4. Conclusions

The solar-driven production of long-term storable and transportable renewable fuels is an efficient decarbonation pathway. This study investigated isothermal chemical looping methane reforming using SrFeO_3-δ_ oxygen carrier porous foam. The thermochemical redox performance was assessed in a solar tubular reactor heated by real concentrated solar irradiation. The impact of the cycling temperature (950–1050 °C), methane concentration in the input stream (5–30% with a constant total flowrate of 0.5 NL/min), and type of oxidizing gas (H_2_O vs. CO_2_) on syngas production performance was unraveled. The increase in temperature and CH_4_ mole fraction chiefly enhanced the syngas production rate in the first step. The syngas yield was also enhanced mainly due to the increase in H_2_ production from the methane cracking reaction. The maximum H_2_ and CO flowrates were about 70 and 25 mL/min at 1000 °C during CH_4_-induced reduction (with 75 mL/min CH_4_ flowrate and 15% mole fraction). The maximum H_2_ flowrate in the first step was increased from 31 mL/min at 950 °C to 100 mL/min at 1050 °C, while the CO flowrate also increased moderately from 11 to 36 mL/min. When the CH_4_ mole fraction was increased from 5% to 30%, the maximum H_2_ flowrate was enhanced from 20 to 88 mL/min, and the CO flowrate increased from 9 to 30 mL/min.

In contrast, the oxidation step was not significantly affected by the experimental conditions (temperature and CH_4_ flowrate). Moreover, the redox performance of the oxygen carrier material was weakly dependent on the nature of oxidizing gas. The total amount of syngas produced including both cycle steps remained stable at a given temperature using either H_2_O or CO_2_ (1363 mL with H_2_O vs. 1373 mL with CO_2_ at 1050 °C). A total syngas yield in the range 660–744 mL/g was achieved at 1000 °C with 15% CH_4_ during reduction, increasing up to 778 mL/g with 30% CH_4_. The global performance of the SrFeO_3-δ_ perovskite foam strongly outperformed the previous values reported to date for ceria.

The thermochemical material performance was not significantly altered during 12 successive cycles. Both the yield and the production rate of syngas in the reduction and oxidation steps remained relatively stable through redox cycles, while the material retained its morphological structure. After the consecutive redox cycles, the material thermochemical stability is confirmed. The proposed SrFeO_3_ redox material thus exhibits good syngas production capacity and cyclability in the solar reactor under real solar irradiation conditions. The considered foam perovskite oxygen carrier is thus suitable for solar-driven chemical looping methane reforming, enabling solar energy storage as valuable chemical fuels. Future work should focus on optimizing the redox performance of the material via fine tuning of experimental conditions while addressing long-term cycling stability.

## Figures and Tables

**Table 1:** Summary of experimental conditions during 12 successive cycles with SFO foam.

Cycle No.	Ar in 1st Step (mL/min)	CH_4_ in 1st Step (mL/min)	Ar in 2nd Step (mL/min)	H_2_O (g/min)/CO_2_ (mL/min) in 2nd Step	T (°C)
1	425	75	500	0.19 g/min	1000
2	425	75	500	0.19 g/min	1000
3	425	75	500	0.19 g/min	950
4	425	75	500	0.19 g/min	1000
5	425	75	500	0.19 g/min	1050
6	425	75	350	150 mL/min	1000
7	425	75	350	150 mL/min	1050
8	425	75	500	0.19 g/min	1000
9	475	25	500	0.19 g/min	1000
10	350	150	500	0.19 g/min	1000
11	350	150	500	0.19 g/min	1000
12	425	75	500	0.19 g/min	1000

## Data Availability

Data will be made available on request.

## Supplementary Materials

The following supporting information can be downloaded at: https://www.mdpi.com/article/10.3390/molecules30051076/s1, Table S1: Overview of the experimental results obtained during 12 successive cycles with SFO foam (1.93 g), Figure S1: XRD analysis of SrFeO_3_ materials before and after thermochemical cycles.
