# Supplementary material for: Chemical Looping CH4 Reforming Through Isothermal Two-Step Redox Cycling of SrFeO3 Oxygen Carrier in a Tubular Solar Reactor
Source: Molecules. 2025 Feb 26;30(5):1076. doi: 10.3390/molecules30051076 (PMC11901619; doi:10.3390/molecules30051076)
Supplement: Supplementary file 1 [file molecules-30-01076-s001.zip › molecules-3483971-supplementary.pdf]

Supplementary Materials

Chemical looping CH_4 reforming through isothermal two-step redox cycling of $SrFeO_3$ oxygen carrier in a tubular solar reactor

Stéphane Abanades [1,*], Xinhe Wang [1,2], Srirat Chuayboon [1,3]

[1] CNRS, Processes, Materials and Solar Energy Laboratory (PROMES-CNRS), 7 Rue du Four Solaire, 66120 Font-Romeu, France; stephane.abanades@promes.cnrs.fr
[2] State Key Laboratory of Multiphase Flow in Power Engineering, Xi'an Jiaotong University, Xi'an, Shaanxi, 710049, China
[3] Department of Mechanical Engineering, King Mongkut's Institute of Technology Ladkrabang, Prince of Chumphon Campus, Chumphon 86160, Thailand

* Correspondence: stephane.abanades@promes.cnrs.fr, (S. Abanades)

Table S1. Overview of the experimental results obtained during 12 successive cycles with SFO foam (1.93 g).

	Cycle #1	Cycle #2	Cycle #3	Cycle #4	Cycle #5	Cycle #6	Cycle #7	Cycle #8	Cycle #9	Cycle #10	Cycle #11	Cycle #12
**Reaction temperature (°C)**	1000	1000	950	1000	1050	1000	1050	1000	1000	1000	1000	1000
**Ar introduced in 1st step (mL/min)**	425	425	425	425	425	425	425	425	475	350	350	425
**CH_4 introduced in 1st step (mL/min)**	75	75	75	75	75	75	75	75	25	150	150	75
**Ar introduced in 2nd step (mL/min)**	500	500	500	500	500	350	350	500	500	500	500	500
**H_2 introduced in 2nd step (g/min)**	0.19	0.19	0.19	0.19	0.19	0	0	0.19	0.19	0.19	0.19	0.19
**CO_2 introduced in 2nd step (mL)/min)**	0	0	0	0	0	150	150	0	0	0	0	0
**H_2 total (mL)**	1130.114	1083.900	1046.113	1046.256	1065.380	503.547	674.637	988.313	837.640	1159.434	1195.451	1032.614
**CO total (mL)**	293.907	283.203	270.300	293.345	288.743	616.856	693.838	282.546	265.530	298.350	299.243	288.905
**CO_2 total (mL)**	11.849	6.973	9.267	6.380	8.689	13.624	4.303	2.959	9.259	6.314	7.246	6.850
**H_2 produced in 1st step (mL)**	716.211	677.172	638.727	651.202	674.862	503.547	674.637	628.155	474.792	784.984	818.825	655.858
**CO produced in 1st step (mL)**	262.249	253.640	240.149	263.140	260.082	202.976	260.346	257.883	239.767	273.691	274.750	262.767
**CO_2 produced in 1st step (mL)**	9.127	4.530	5.223	3.840	5.898	13.624	4.303	0.813	6.573	2.696	4.025	3.561
**C_nH_m produced in 1st step (mL)**	3.173	1.829	5.589	1.839	0.878	2.008	1.274	1.537	0.441	38.220	56.552	2.683
**CH_4 remained in 1st step (mL)**	1463.935	1175.990	2562.764	1286.083	645.396	1225.901	650.166	1135.277	567.891	2116.894	2689.205	1288.272
**Maximum CH_4 conversion (%)**	46.7	44.1	21.5	42.0	62.4	36.4	53.7	36.5	62.8	26.0	27.5	38.7
**H_2 produced in 2nd step (mL)**	413.903	406.728	407.386	395.054	390.518	0.000	0.000	360.158	362.849	374.450	376.625	376.756
**CO produced in 2nd step (mL)**	31.658	29.563	30.150	30.206	28.661	413.880	433.492	24.664	25.762	24.659	24.494	26.138
**CO_2 produced in 2nd step (mL)**	2.723	2.443	4.044	2.540	2.791	-	-	2.145	2.686	3.618	3.221	3.289
**H_2 purity in 2nd step**	0.923	0.927	0.923	0.923	0.925	0.000	0.000	0.931	0.927	0.930	0.931	0.928
**CH_4 reacted (mL)**	305.756	290.176	279.567	299.726	297.432	265.398	341.622	285.505	274.789	304.663	306.489	295.754
**CO selectivity**	0.961	0.976	0.967	0.979	0.971	0.765	0.762	0.990	0.966	0.979	0.976	0.977
**Syngas selectivity**	1.067	1.069	1.048	1.017	1.048	0.887	0.912	1.034	0.867	1.158	1.189	1.035
**Coke selectivity**	0.112	0.110	0.122	0.109	0.106	0.184	0.225	0.094	0.104	0.093	0.090	0.099
**Mass of coke (g)**	0.017	0.016	0.017	0.016	0.016	0.024	0.038	0.013	0.014	0.014	0.014	0.015
**Ratio of H_2 to CO in 1st step**	2.731	2.670	2.660	2.475	2.595	2.481	2.591	2.436	1.980	2.868	2.980	2.496

**Ratio of H_2 to CO in total**	3.845	3.827	3.870	3.567	3.690	0.816	0.972	3.498	3.155	3.886	3.995	3.574
**Oxygen consumed (mL)**	298.755	271.762	261.041	278.500	283.675	257.474	277.559	261.136	266.061	284.474	290.849	277.010
**Oxygen consumed (mol)**	0.012	0.011	0.011	0.012	0.012	0.011	0.012	0.011	0.011	0.012	0.012	0.012
**Oxygen recovered (mL)**	376.800	372.280	369.148	359.768	356.275	316.286	279.546	331.204	331.716	342.555	345.690	344.041
**Oxygen recovered (mol)**	0.016	0.015	0.015	0.015	0.015	0.013	0.012	0.014	0.014	0.014	0.014	0.014

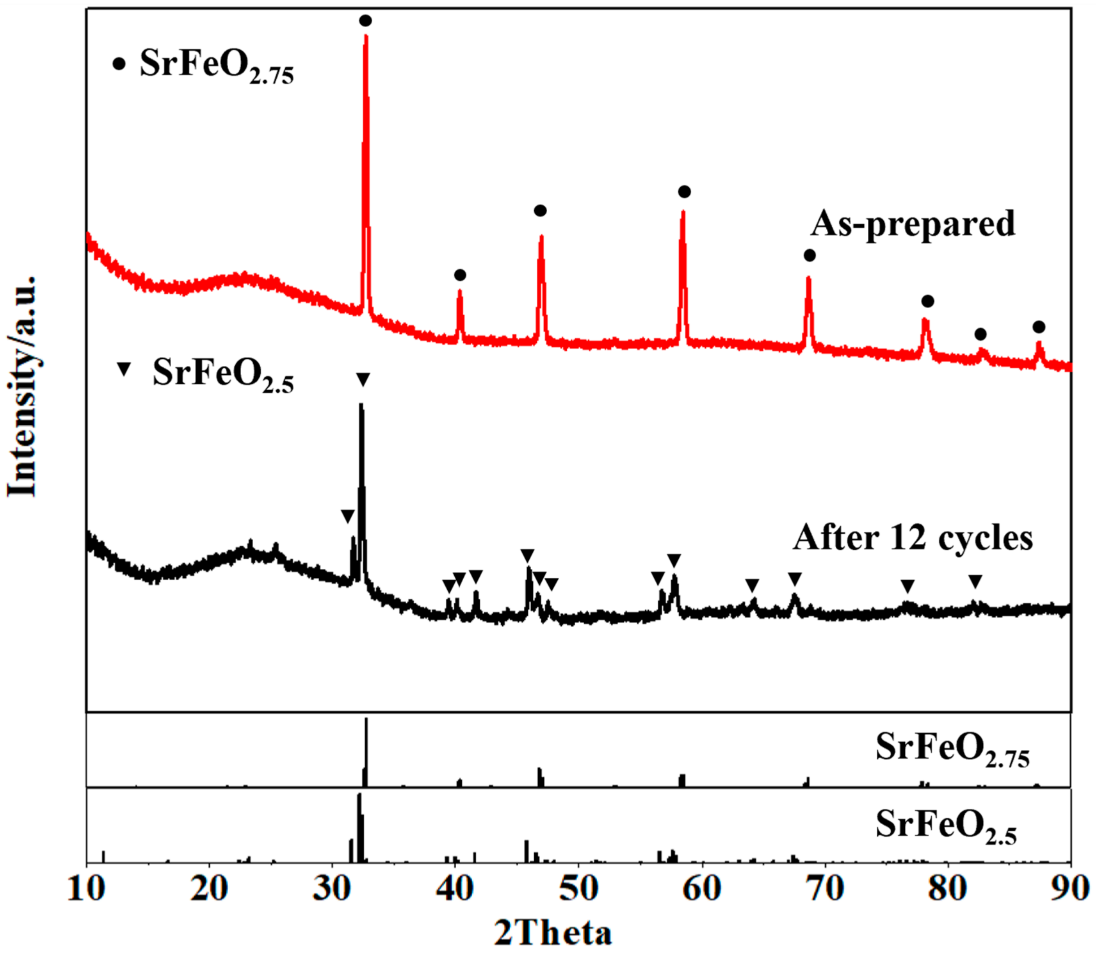

Figure S1: XRD analysis of $SrFeO_3$ materials before and after thermochemical cycles.
